# Estimating vastus lateralis muscle volume from a single ultrasound image

**DOI:** 10.1038/s41598-025-11437-5

**Published:** 2025-08-18

**Authors:** Moritz Eggelbusch, Guido Weide, Michael Tieland, Renske N. Vergeer, Luuk Vos, Jantine van den Helder, Stephan van der Zwaard, Richard T. Jaspers, Peter J. M. Weijs, Rob C. I. Wüst

**Affiliations:** 1 Laboratory for Myology, Department of Human Movement Sciences, Faculty of Behavioural and Movement Sciences, Vrije Universiteit Amsterdam, Amsterdam Movement Sciences, Amsterdam, The Netherlands; 2 Faculty of Health, Sport and Physical Activity, Centre of Expertise Urban Vitality, Amsterdam University of Applied Sciences, Amsterdam, The Netherlands; 3 Present Address: Professorship of Exercise Biology, Department Health and Sport Sciences, TUM School of Medicine and Health, Technical University of Munich, Munich, Germany; 4 Institute for Physical Activity and Nutrition, School of Exercise and Nutrition Sciences, Deakin University, Geelong, VIC Australia; 5 Department of Cardiology, Amsterdam University Medical Centers, University of Amsterdam, Amsterdam, The Netherlands; 6 Department of Biomedical Engineering and Physics, Amsterdam University Medical Centers, University of Amsterdam, Amsterdam, The Netherlands

**Keywords:** Ultrasound, Skeletal muscle volume, Anatomical cross-sectional area, Skeletal muscle thickness, Echocardiography, Ultrasound, Skeletal muscle

## Abstract

The assessment of skeletal muscle volume is valuable for fundamental research and clinical practice, but remains limited in larger cohorts due to its time-consuming nature. Here, we developed a method to accurately estimate vastus lateralis (VL) muscle volume based on a single measurement of anatomical cross-sectional area (ACSA) or tissue thickness. Sixty-nine healthy participants (20–91 years) volunteered. In a subgroup (*n* = 34) we measured VL volume and ACSAs at 10% intervals along the muscle length to derive a VL muscle shape factor. We subsequently estimated VL volume by multiplying this muscle shape factor with muscle length and a single measure of ACSA at 50% muscle length (ACSA_VL50%_) or an estimated ACSA_VL50%_ from a single ultrasound scan of tissue thickness in an independent cohort (*n* = 35). VL muscle shape factor was determined by integrating a fourth-order polynomial of muscle length and ACSA, and was dependent on muscle size. Estimating muscle volume had a high accuracy (R²=0.976, CCC = 0.987), low bias and error (< 8.5%) in both the main cohort and an independent validation group. Estimating muscle volume from stitching 2D images at 50% muscle length or estimating ACSA with a geometric model explained 91–95% of variance in measured volumes, with high accuracy and concordance correlation coefficients. VL muscle volume can be estimated by multiplying a muscle shape factor with muscle length and ACSA_VL50%_ from a single ultrasound image. We present a novel, cost-effective, rapid, yet accurate assessment of VL muscle mass for (large-scale) studies and clinical practice.

## Introduction

During critical illness, patients experience fatigue and accelerated muscle atrophy that can persist for months post-discharge^1–3^. Critically ill patients in the intensive care unit (ICU) can lose up to 1 kg of muscle mass per day^4^with approximately 2% of skeletal muscle mass being lost daily in the first week of ICU admission^5^. This severe muscle wasting significantly contributes to ICU-acquired weakness, a common complication of critical illness. Additionally, low muscle mass was the main risk factor for mortality in mechanically ventilated critically ill patients^6^. Muscle power measured before ICU discharge has been identified as a strong independent predictor of functional recovery^7^. Muscle power and changes in echogenicity index of the rectus femoris, but not muscle strength, were the strongest predictors of ICU-acquired weakness. Multivariate regression analyses identified muscle power, age, and ICU length of stay as significant predictors of functional performance at discharge^7^. Determining skeletal muscle volume therefore provides crucial information for explaining physical performance in various populations as well as disease outcome and survival in ICU patients^6,7^.

Muscle mass is not only an important tissue for glucose uptake and the regulation of blood glucose homeostasis^8^,but low skeletal muscle mass is also associated with poor clinical outcomes in cancer patients^9^. Despite this, assessing muscle volume is not common practice in hospitalized patients due to measurement complications, costs and equipment availability. The non-invasive assessment of skeletal muscle morphology is valuable for understanding muscle force- and power-generating capacity in both fundamental research and clinical practice. While maximal voluntary force and muscle volume are typically positively correlated^10^,variations in muscle length in cross-sectional analyses result in individuals with similar anatomical cross-sectional area (ACSA) having significantly different muscle volumes. This correlation between muscle volume and power-generating capacity is well-established, not only in young healthy individuals^11^,but also in older populations^12^.

Common methods for assessing lean muscle mass or volume include bioelectrical impedance analysis, ultrasound or advanced imaging techniques such as dual-energy X-ray absorptiometry, magnetic resonance imaging (MRI) or computed tomography, which are often unavailable, too costly, invasive or of limited validity^13–15^. With the aid of a motion capture system, 3D ultrasound techniques have been developed to reconstruct muscle volume and morphology, providing a viable, and accurate alternative for the quantification of skeletal muscle mass^16,17^. Mean differences of 3D ultrasound vs. MRI-assessed muscle volumes ranged from − 0.13 to 1.5%, with intra-class correlation coefficients of 0.98 or higher^18–20^. However, position tracking devices are often unavailable in hospitals and require specific expertise. Application in large clinical cohorts and practice is further limited due to the time-consuming nature of these measurements and analyses. Measurements of the ACSA present a reliable, cost- and time-effective alternative for assessing skeletal muscle mass^21–24^. However, two-dimensional measures (ACSA) do not always reflect the three-dimensional volume of a muscle, since muscles can have different lengths and non-uniform shapes. As such, a new method is required that is feasible in clinical practice, requires minimal equipment and training, and provides a reliable estimate of muscle volume without the need for time-consuming or cost-intensive procedures.

To overcome these limitations, we aimed to develop an ultrasound-based estimation approach of muscle volume based on a muscle shape factor, muscle length and a single vastus lateralis (VL) ACSA measurement at 50% muscle length, similar as previously performed using MRI^15,25^. The VL is the most superficial and largest knee extensor muscle in both athletes and non-athletes^26^,crucial for power production^27^,and the VL is the predominant muscle in biopsy and imaging studies. To reduce measurement complexity and time, we tested a novel approach where muscle thickness from a single ultrasound image was used to estimate VL ACSA, and subsequently estimated VL volume. VL volume estimations were compared against 3D ultrasound-measured muscle volumes to test their accuracy, and the estimation approach was validated in an independent cohort. We hypothesized that our estimated VL volumes show substantial concordance with 3D ultrasound-acquired volumes.

## Methods

### Participants

Overall, 69 healthy volunteers (age range: 20–91 years, 23 females) participated in this study. The study was conducted according to the principles of the Declaration of Helsinki and was approved by the departmental ethics committees of the Vrije Universiteit and the Amsterdam University Medical Centers (Amsterdam, the Netherlands). Prior to participation, participants were informed about the experimental procedures of the study and provided written informed consent.

### Muscle volume assessed by 3D ultrasound

**Fig. 1:**
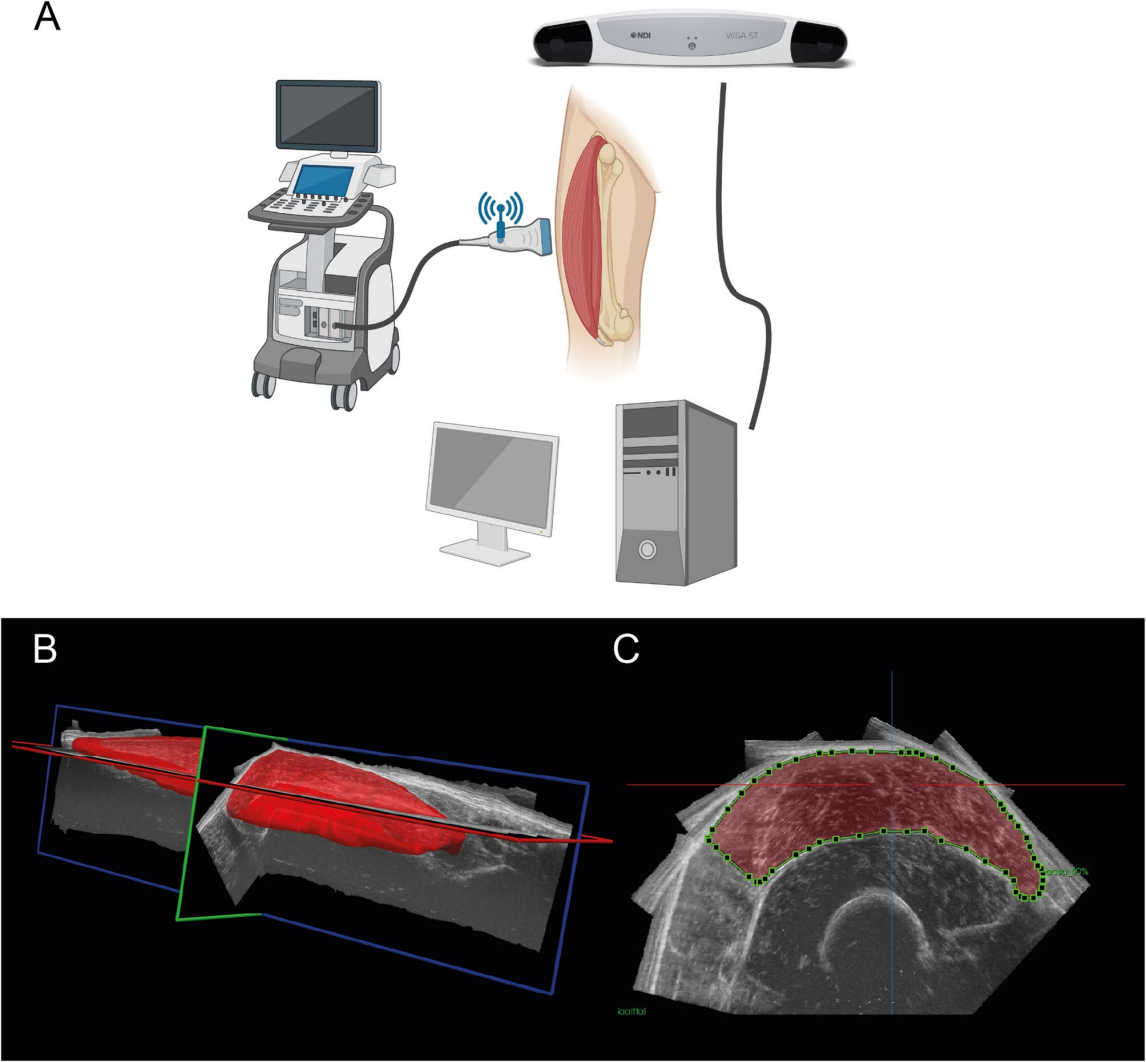
3D ultrasound methodology. (**A**) Ultrasound scans of the vastus lateralis were collected in longitudinal direction distally to proximally, covering the entire muscle belly. Location and orientation of the ultrasound probe were collected over time using a cluster marker set attached to the ultrasound probe, traced by a pose tracking device, enabling the construction of a 3D vastus lateralis muscle. (**B**) Representative example of a 3D ultrasound image reconstruction. (**C**) A segmented vastus lateralis anatomical cross-sectional area at 50% muscle length in the transversal plane using reconstructed images.

Muscle morphology of the vastus lateralis muscle was obtained using 3D ultrasound imaging (Fig. 1), as described previously^17^. 3D ultrasound acquisitions of the right vastus lateralis were obtained with the participant seated in 85° hip flexion and 60° knee flexion. To prevent displacement or rotation of the legs, the lower leg was strapped and secured to the bench. Ultrasound images were acquired in B-mode (30 Hz) using a 5-cm linear probe attached to an ultrasound machine (Arietta Prologue, Hitachi L-55, Hitachi Ltd., Tokyo). Location and orientation of the ultrasound probe were collected over time using a cluster marker set attached to the ultrasound probe, traced by a pose tracking device (Polaris Vega, NDI, Waterloo, Canada). Ultrasound sweeps of the vastus lateralis were collected in longitudinal direction, starting from the lateral muscle border, scanning from the distal to proximal end of the muscle belly with the probe in transverse orientation. Subsequent sweeps were taken medially from the previous sweep with a ~ 1 cm overlap. An external trigger marked the start of each scan and was used to synchronize position data with the ultrasound B-mode images to enable construction of a 3D voxel array using customized software (MatLab; MathWorks, Natick, MA, USA). The ultrasound voxel array was subsequently analyzed using 3D image-processing software (The Medical Imaging Interaction Toolkit v2023.04; German Cancer Research Center, Heidelberg, Germany). After identifying proximal and distal end of the vastus lateralis muscle as the greater trochanter and distal musculotendinous junction, respectively, muscle volume was determined by manually segmenting cross-sections at regular (5–10%) intervals, evenly distributed along the muscle belly length, while excluding adipose tissue and connective tissue incursions. Interactive interpolation of segmented cross-sections was then applied, and manually checked along all cross-sections, to calculate muscle volume from the 3D ultrasound voxel array (where voxel size was 0.2 × 0.2 × 0.2 mm^3^). Our previous work showed high intra-rater and test-retest reliability of the 3D ultrasound^17,28^.

### Muscle cross-sectional area by 2D B-mode ultrasound image stitching

We defined the distal end of the muscle belly as the endpoint for length measurements and ACSA positioning, not the anatomical insertion of the vastus lateralis. Proximal and distal end of the vastus lateralis muscle (i.e., greater trochanter and distal musculotendinous junction) were identified through palpation, confirmed using B-mode ultrasound, and marked on the skin with the participant seated in 85° hip flexion and 60° knee flexion. The medial and lateral border of the vastus lateralis were identified at 50% of muscle length by ultrasound and marked on the skin. A measurement tape was firmly attached around the participant’s leg to divide the width of the vastus lateralis into 3 cm sections, and to ensure consistent probe placement and full muscle capture. Consecutive 2D B-mode ultrasound images were taken, moving from the medial to the lateral muscle border with a standardized overlap between individual images. Acquired images were merged in Microsoft PowerPoint and the vastus lateralis ACSA at 50% muscle length was outlined using the polygon tool in the composite image in ImageJ.

### Muscle thickness and width by ultrasound

A single ultrasound image was taken at 50% of the muscle length in the mid-section of the muscle. Briefly, 50% VL muscle length and medial and lateral muscle borders were identified as described above. The projection of the VL on the skin was measured as the distance from medial to lateral VL border (Width_VL_) using a tape measure. The ultrasound probe was placed in the middle of the muscle, and a single ultrasound image in B-mode was taken.

### Muscle volume estimation models

#### Muscle volume regression model

In reconstructed 3D ultrasound scans, the proximal and distal end of the vastus lateralis muscle were identified as the greater trochanter and distal musculotendinous junction, respectively. After measuring the vastus lateralis muscle length, transverse segmentations of the muscle were performed manually in the axial plane at 10% intervals along the length of the muscle. Relative ACSAs were plotted against relative muscle lengths and the data was fitted with a fourth-order polynomial^15^. Interpolation of this polynomial from 0 to 1 yielded a “muscle shape factor” (λ). Vastus lateralis muscle volume was estimated by multiplying the muscle shape factor λ with total muscle length and ACSA_VL50%_:

1 $$\:{Volume}_{VL}=\:{ACSA}_{VL50\%}*{Length}_{VL}*\:\lambda\:\:$$

#### Muscle volume estimation by a single ACSA measure

3D ultrasound-segmented ACSA_VL50%_ as well as “stitched” ACSA_VL50%_ were used to estimate vastus lateralis muscle volume employing Eq. 1, and estimates plotted against 3D ultrasound-assessed muscle volume.

#### A muscle thickness-based approach to estimate cross-sectional area and volume

**Fig. 2:**
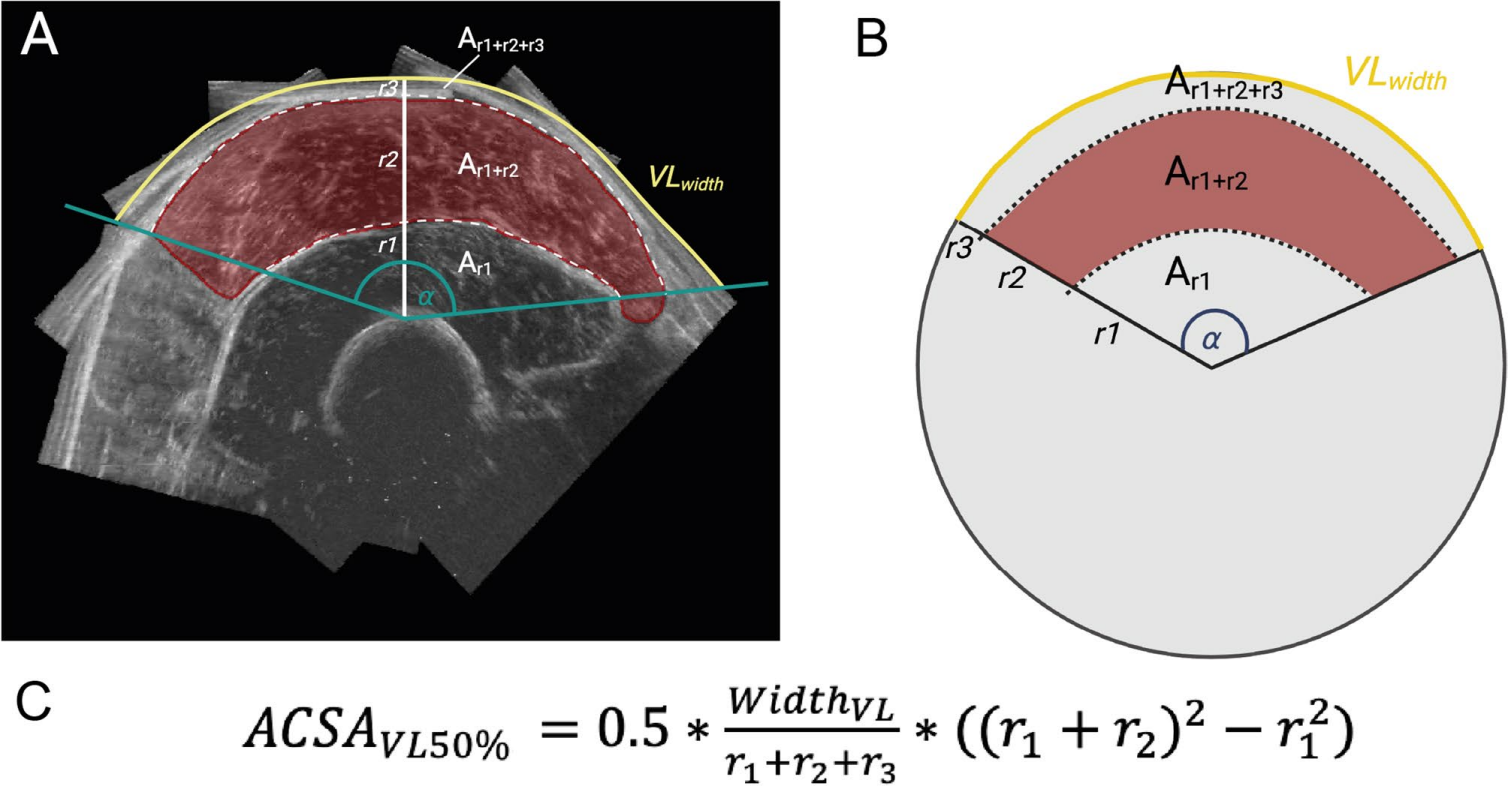
Mathematical simplification of VL ACSA estimation based on thickness measurements. (**A**) Representative transverse reconstruction of the VL anatomical cross-section in red, with its shape similar to a circular sector. (**B**,**C**) A sector of a circle is calculated by πr^2^ x α/360, and the VL projection on the skin (Width_VL_; from medial to lateral muscle border, depicted in yellow) is calculated by Width_VL_ = 2πr x α/360. Rearranging both formulas using different radiuses 1–3 (where r_1_ = vastus intermedius thickness, r_2_ = vastus lateralis thickness and r_3_ = epidermis and subcutaneous fat layer) results in equation (**C**), where ACSA_VL_ can be estimated using the assessment of r_1_, r_2_, r_3_ and Width_VL_.

The determination of ACSA_VL50%_ was further simplified by using muscle thickness (measured as described above) in combination with a measure of VL width (Width_VL_). Given that the shape of the VL ACSA can be modelled as a sector of a circle (Fig. 2A, B), we used generic geometrical equations to estimate ACSA from a single ultrasound image. Vastus intermedius (r_1_) and VL (r_2_) thickness as well as the epidermis and subcutaneous fat layer (r_3_) were measured in ImageJ, drawing vertical lines from the most superficial part of the femur to the deep aponeurosis, from the deep to the superficial aponeurosis, and from the superficial aponeurosis of the VL to the skin surface, respectively (cf., Figs. 2 and 5). Figure 2 provides a mathematical explanation. In short, a sector of a circle (A_S_) is calculated by:

2 $$\:{A}_{S}=\:{\pi\:r}^{2}*\frac{\alpha\:}{360},$$

with r being the distance from femur to skin surface (i.e., r = r_1_ + r_2_ + r_3_ in Fig. 2A, B), and α the angle between the medial and lateral VL border with respect to the femur. A circular arc, i.e., Width_VL_ (the skin distance from medial to lateral border of the VL, Fig. 2), is calculated by:

3 $$\:{Width}_{VL}=2\pi\:r*\frac{\alpha\:}{360}.$$

**Fig. 3:**
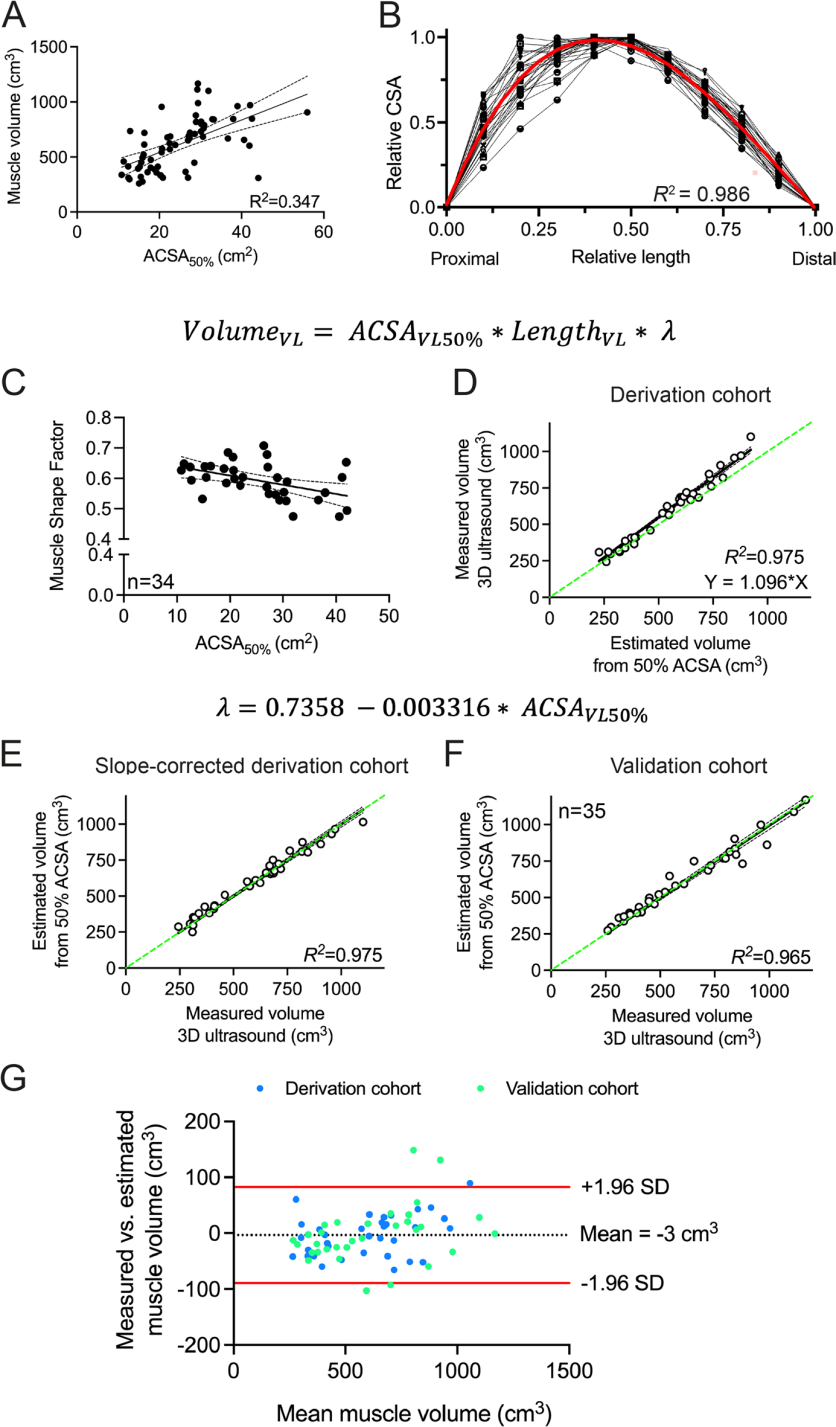
An estimation for the assessment of vastus lateralis muscle volume. (**A**) Muscle cross-sectional area (ACSA) at 50% of muscle length correlated with muscle volume (R^2^ = 0.347), with large 95% confidence intervals for slope (14.7 [9.6–19.8] cm) and intercept (248 [112–384] cm^3^). (**B**) Anatomical cross-sectional areas (ACSAs) measured at 10% intervals along the length of the vastus lateralis (VL) were expressed relative to the maximum ACSA per individual and plotted against the corresponding relative muscle length in 34 participants. A fourth-order polynomial was derived from the dataset, and integration over the relative muscle length and ACSA resulted in a constant “muscle shape factor” ($\:\lambda\:$), which can be used to estimate vastus lateralis muscle volume taking into account a single ACSA measurement at 50% muscle length and the entire muscle length$\:.$ (**C**) Individual muscle shape factors correlated moderately to muscle size. (**D**) Comparing measured and estimated VL muscle volume based on the equation in (**A**) showed a very strong correlation, albeit with a correction factor of 9.6%. (**E**) Slope-corrected muscle volume estimations closely resembled absolute muscle volumes. (**F**) Accuracy of vastus lateralis muscle volume estimation holds true in an independent validation cohort (*n* = 35). (**G**) Bland-Altman analysis of both cohorts showed a bias of -3 ± 44 cm^3^with 95% limits of agreement ranging from − 82 to 83 cm^3^.

Solving Eq. 3 for angle α yields:

4 $$\:\alpha\:=\frac{180}{\pi\:}*\frac{{Width}_{VL}}{{r}_{1}+{r}_{2}+{r}_{3}}.$$

Equation 2 is used to calculate the total vastus area (r_1_ + r_2_-related area), and vastus intermedius area (r_1_-related area) individually. Combing Eq. 2 + 4, vastus lateralis muscle volume is obtained from subtracting the vastus intermedius volume (r_1_-related area) from the total vastus area:

5 $$\:{ACSA}_{VL50\%}=0.5*\frac{{Width}_{VL}}{{r}_{1}+{r}_{2}+{r}_{3}}*({({r}_{1}+{r}_{2})}^{2}-{r}_{1}^{2}).$$

As such, the only required measurements for Eq. 5 are Width_VL_ (measured by a tape measure on the skin) and tissue thickness measurements (r_1_, r_2_ and r_3_; measured in a single ultrasound image), which were described above. Subsequently, estimated ACSA_VL50%_ was used to estimate VL muscle volume employing Eq. 1.

### Statistics

The 3D ultrasound-based assessment of VL muscle volume was regarded as ground truth^17^. Data was checked for normality by the Shapiro-Wilk-test. Group differences were tested using independent Student’s t-tests, or Mann–Whitney U test if data was not normally distributed. Linear regression and Pearson’s correlation analysis were used to explore the degree of association between muscle thickness- and 2D ultrasound-derived estimated parameters with respect to the ground truth. To assess the level of agreement between estimated and measured parameters, a Bland-Altman analysis was performed to evaluate the bias of estimated values and to determine the 95% limits of agreement. Standard error of the estimate (SEE), root mean square error (RMSE), and Lin’s concordance correlation coefficient (CCC)^29^ were calculated as final accuracy measures of estimated parameters. CCC values were interpreted according to McBride^30^ with $\:\rho\:$_c_ values below 0.90 being considered poor, between 0.90 and 0.95 considered moderate, between 0.95 and 0.99 as substantial, and above 0.99 as almost perfect. All statistical analyses were performed using GraphPad Prism version 10 and Rstudio (RStudio: Integrated Development for R. RStudio, PBC, Boston, MA, USA). Data is presented as mean ± standard deviation, with the alpha level for statistical significance set to *p* < 0.05.

## Results

### Regression model and muscle shape factor

Our 3D ultrasound setup is depicted in Fig. 1, together with a typical 3D ultrasound image reconstruction and an example of a segmented vastus lateralis (VL) anatomical cross-sectional area (ACSA) in the transversal plane (Fig. 1A–C). Our cohort of 69 participants had a broad age range (20–91 years; participant characteristics in Table 1).

**Table 1:** Baseline characteristics in male and female participants.

	Males (*n* = 46)	Females (*n* = 23)
Age (years)	42 ± 18	52 ± 25
Height (cm)	185 ± 9	167 ± 9
Weight (kg)	84.6 ± 13.2	70.0 ± 13.8
BMI (kg/m^2^)	23.8 ± 2.9	25.0 ± 3.4
Muscle length (cm)	38.6 ± 2.6	34.6 ± 2.8

Participant characteristics (mean ± standard deviation). Data from four cadavers were not included.

For the regression model, we used data from 34 participants (see Table 1) with an average 3D ultrasound-acquired VL muscle volume of 632 ± 216 (range: 244–1101) cm^3^. Anatomical cross-sectional area at 50% of muscle length correlated with muscle volume, albeit with a low R^2^ (0.347) and large 95% confidence intervals for slope and intercept (Fig. 3A), highlighting the need to incorporate muscle length and shape factor to improve volume estimation. Only including muscle length significantly increased the correlation between estimated (muscle length x ACSA_VL50%_) and real volumes (*R*^*2*^ = 0.932), albeit with a large overestimation of muscle volumes. This requires the addition of a muscle shape factor. Therefore, we reconstructed the VL muscle using ACSAs at 10% intervals along the relative muscle length, and relative to the individual maximum ACSA (Fig. 3B). From this, we derived a fourth-order polynomial per individual, with *R*^*2*^ = 0.986 ± 0.016 (Fig. 3B). Interpolation of the individual polynomial equations over the relative muscle length (0–1) and relative ACSA (0–1) resulted in a “muscle shape factor” of 0.593 ± 0.059 (Fig. 3B):

**Fig. 4:**
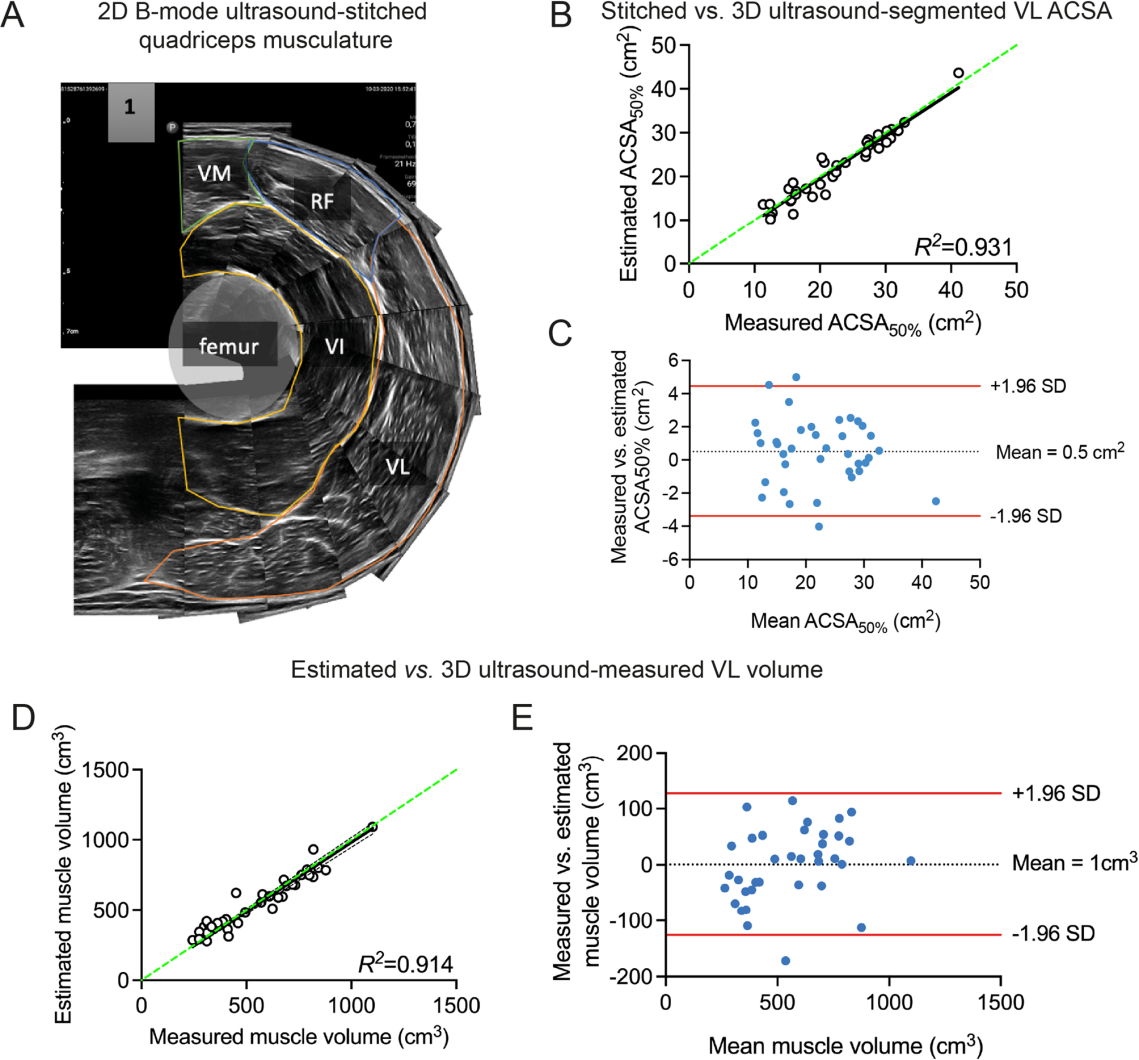
Ultrasound image stitching for the anatomical cross-section and subsequent estimation of vastus lateralis muscle volume. (**A**) B-mode ultrasound image stitching resulted in a composite image that was compared with the segmented VL ACSA in the transversal plane of 3D reconstructed muscles. (**B**–**E**) Regression analyses and Bland-Altman plots for the comparison of stitched vs. 3D ultrasound-acquired VL ACSA. (**B**,**C**) and for estimated vs. measured muscle volume (**D**,**E**).

6 $$\:\lambda\:={\int\:}_{0}^{1}+4.401\left(\pm\:7.554\right){x}^{4}-5.814\left(\pm\:16.182\right){x}^{3}-2.816\left(\pm\:11.003\right){x}^{2}+4.232\left(\pm\:2.333\right)x-0.011(\pm\:0.047)dx=0.593\pm\:0.059.$$

This muscle shape factor $\:\lambda\:$ can be interpreted as a correction factor of the VL shape relative to the cylinder determined by $\:{ACSA}_{VL50\%}*{Length}_{VL}$. We tested whether the muscle shape factor $\:\lambda\:$ is dependent on muscle size, considering that larger muscles may have a different shape compared to smaller ones. Individual muscle shape factors correlated moderately to muscle size (*R*^*2*^ = 0.221, *p* = 0.005, Fig. 3C), meaning that larger muscles had proportionally different shapes. The muscle shape factor was derived by considering the intercept and slope of the muscle shape against size association via the following equation:

7 $$\:\lambda\:=\:0.6693\: - \:0.00302\:\text{*}{ACSA}_{VL50\%}.$$

The 95% confidence interval of the intercept is 0.615 to 0.724, and of the slope − 0.005060 to − 0.0009753. The measured muscle volume correlated well to the estimated volume (*R*^*2*^ = 0.975), albeit with a correction factor of 9.6% (Fig. 3D). As such, we adjusted the derivation equation for the muscle shape factor accordingly (combining Eq. 7 and the correction factor of 9.6%), to:

8 $$\:\lambda\:\:=\:0.7358\: - \:0.003316\text{*}{ACSA}_{VL50\%}.$$

Indeed, the slope-corrected estimation of muscle volume closely resembled the absolute muscle volumes, with narrow 95% confidence intervals (*R*^*2*^ = 0.975, Fig. 3E). Estimated VL muscle volume was on average 600 ± 214 cm^3^with an absolute difference of 31 ± 21 cm^3^ vs. measured volume (596 ± 227 cm^3^; 6.0 ± 5.0% relative difference). Regression analysis showed that the estimation equation explained 97.5% of variance in measured muscle volume, with a standard error of the estimate (SEE) of 37 cm^3^ (6.2%), substantial concordance (CCC = 0.986; Fig. 3E) and a root mean square error (RMSE) of 37 cm^3^ (Table 2).

**Table 2:** Validation of 2D ultrasound- and muscle thickness-based estimates against 3D ultrasound.

	3D ultrasound vs. estimated VL volume (3D ultrasound-segmented ACSA) (cm^3^)	3D ultrasound vs. estimated VL volume in validation cohort (cm^3^)	3D ultrasound-segmented ACSA vs. 2D stitched ACSA (cm^2^)	3D vs. stitched ACSA_50%_-estimated VL volumes (cm^3^)	3D ultrasound-segmented ACSA vs. tissue thickness-estimated ACSA (cm^2^)	3D ultrasound volume vs. tissue thickness-estimated volume (cm^3^)
Number of participants	34	35	38	38	38	38
Mean ± SD (measured vs. estimated)	596 ± 227 vs. 600 ± 214 cm^3^	606 ± 259 vs. 608 ± 241 cm^3^	22.49 ± 7.32 vs. 21.96 ± 7.62	562 ± 218 vs. 557 ± 195	22.4 ± 7.3 vs. 22.6 ± 8.0	562 ± 218 vs. 558 ± 202
Bias ± SD	− 4 ± 37	− 2 ± 50	0.54 ± 2	2 ± 65	0.5 ± 2.4	1 ± 50
LoA	[− 76cm^3^ 69cm^3^]	[− 101cm^3^ 95cm^3^]	[− 3.4cm^2^ 4.5cm^2^]	[− 125cm^3^ 129cm^3^]	[− 4.2cm^2^ 5.1cm^2^]	[− 97cm^3^ 99cm^3^]
*R* ^*2*^	0.975	0.965	0.931	0.914	0.921	0.948
SEE (%)	37.4 cm^3^ (6.2%)	50.4 cm^3^ (8.3%)	1.9 cm^2^ (8.7%)	58.7 cm^3^ (10.5%)	2.5 cm^2^ (11%)	47.0 cm^3^ (8.4%)
CCC	0.986	0.980	0.962	0.951	0.951	0.972
RMSE	36.9 cm^3^	52.0 cm^3^	2.2 cm^2^	76.4 cm^3^	2.4 cm^2^	49.7 cm^3^

Comparison of 3D ultrasound-measured vastus lateralis (VL) muscle volume and anatomical cross-sectional area (ACSA) vs. 2D ultrasound and muscle thickness measurements and estimations. Bias, limits of agreement (LoA), standard error of the estimate (SSE), Lin’s concordance correlation coefficient (CCC) and root mean square error (RMSE) are presented for each respective comparison.

To validate our estimation equation for VL muscle volume, we applied Eqs. 1 and 8 in an independent validation group of 35 adult individuals (22 males, age: 47 ± 21 years and height: 176 ± 9 cm; with measured VL volume of 606 ± 259 cm^3^*p* = 0.99, Fig. 3F). Mean estimated muscle volume was 608 ± 241 cm^3^accounting for 96.5% of variance in measured volume (Fig. 3F), with an absolute difference of 35 ± 35 cm^3^ vs. measured volume (6.1 ± 5.0%), an SEE of 50.4 cm^3^ (8.3%) and substantial concordance (CCC = 0.980). Bland-Altman analysis of both cohorts showed a bias of -3 ± 44 cm^3^with 95% limits of agreement (-82 to 83 cm^3^) equivalent to -13.5–13.7% of the mean measured volume (Fig. 3G; Table 2).

Subgroup analysis of the combined derivation and validation cohorts for males versus females, matched for ACSA_VL50%_, revealed no significant differences in the muscle shape factor (*p* = 0.392). Also, when participants were divided into subgroups based on the median age of 36 and matched for ACSA_VL50%_, the muscle shape factor did not differ significantly between younger and older individuals (*p* = 0.214).

### A 2D ultrasound image stitching approach

To simplify the approach to estimate muscle volume, we first determined the maximal ACSA along the muscle length. We used a finer resolution (i.e., 1% increments) of the 3D muscle volume assessments to provide a more detailed profile of ACSA distribution along the VL length. We performed this analysis on 21 participants and found that the maximal ACSA was located at 47 ± 10% of the muscle belly length (from proximal), albeit with some inter-individual variability (from 32 to 56%). In 16/27 cases, maximal ACSA was at 50% when assessing with 10% increments. To simplify this, we measured muscle ACSA at 50% of muscle length in 38 participants (28–56 years) with an ultrasound image stitching method, where muscle length was measured on the skin surface, and compared this to our 3D approach (Fig. 4A). Stitched VL ACSAs (22.0 ± 7.5 cm^2^) explained 93% of variance in segmented ACSAs of reconstructed 3D ultrasound images (22.4 ± 7.3 cm^2^; Fig. 4B). Bland-Altman analysis showed a small bias of 0.5 ± 2.0 cm^3^with 95% limits of agreement (-3.4 to 4.5 cm^2^) corresponding to -15–20% of the mean ACSA, and a low SEE (1.9 cm^2^; 8.7%) with substantial concordance (0.962, Fig. 4C; Table 2). Estimating VL muscle volume using stitched VL ACSAs as input for Eqs. 1 and 8 resulted in 557 ± 195 cm^3^with a bias of 2 ± 65 cm^3^ vs. measured muscle volumes (562 ± 218 cm^3^*R*^*2*^ = 0.914, SEE = 58 cm^3^CCC = 0.951; RMSE 76 cm^3^; Fig. 4D, E; Table 2).

**Fig. 5:**
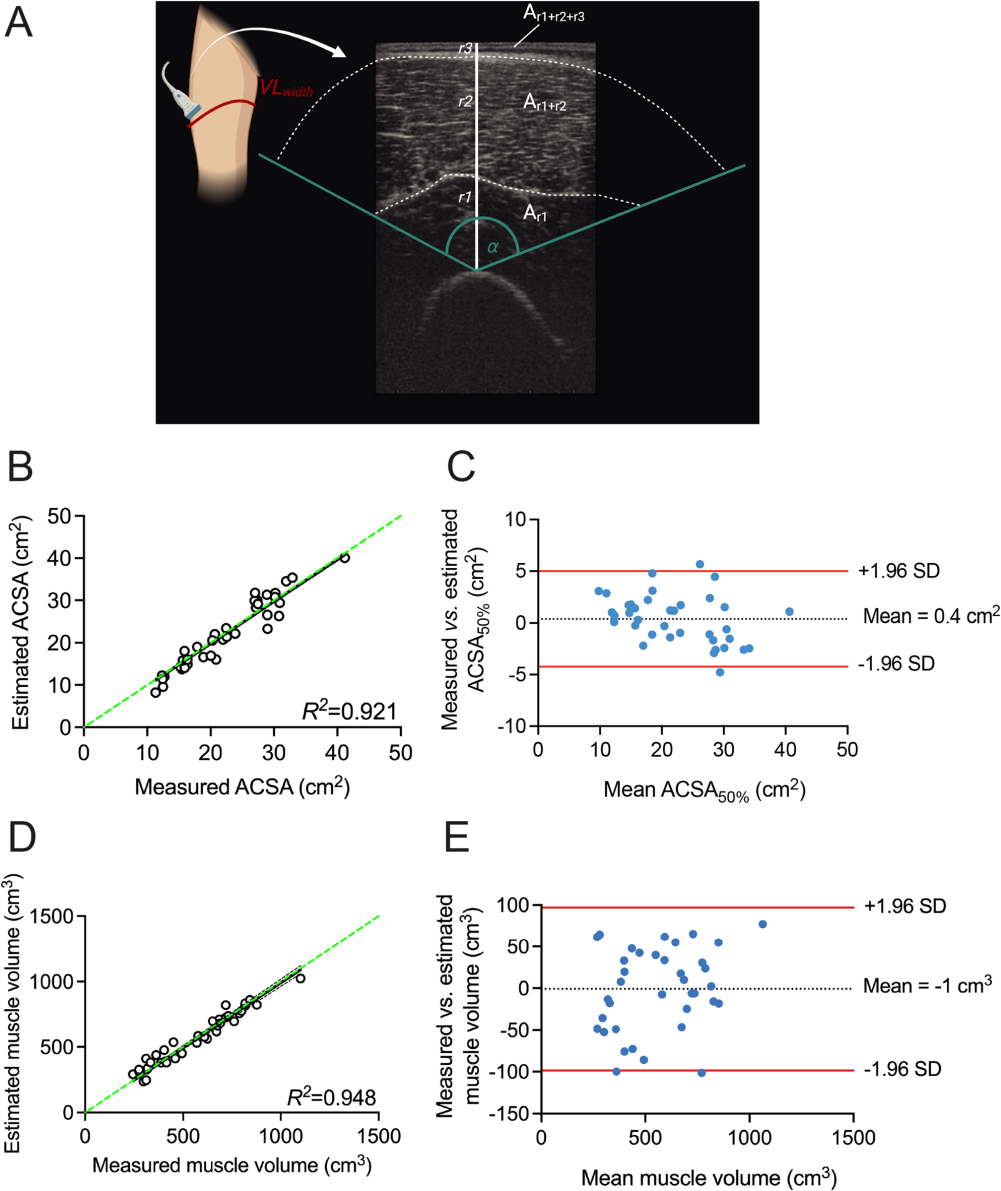
Validation of estimating vastus lateralis anatomical cross-sectional area for the determination of vastus lateralis muscle volume. (**A**) Vastus lateralis (VL) width, measured on the participant’s skin, and muscle thickness (r_1_, r_2_, r_3_) measures derived from a single ultrasound image were used to estimate VL anatomical cross-sectional area at 50% (ACSA_50%_; see Fig. 5 & Eq. 5). (**B**,**C**) Regression analysis and Bland-Altman plots were used to assess the accuracy of estimated ACSAs vs. measured ACSAs. (**D**,**E**) Estimated ACSAs were used to predict VL muscle volume using Eq. 1, and results were compared against measured volumes by regression and Bland-Altman analyses.

### Estimation and validation of vastus lateralis anatomical cross-sectional area

Given that VL ACSA resembles that of a sector of a circle (see “Methods”, Fig. 2A, B), we hypothesised that VL ACSA can be estimated using generic geometrical equations with input derived from a single ultrasound image. To validate our simplified mathematical estimation method for VL ACSA (Eq. 5, cf. Fig. 2), the required input parameters $\:{Width}_{VL}$ and tissue thickness (r_1_, r_2_, r_3_) were measured (Fig. 5A). Using Eq. 5 to estimate ACSA_VL50%_ yielded an average of 22.6 ± 8.0 cm^2^ explaining 92% of variance in 3D ultrasound-measured ACSA_VL50%_ (22.4 ± 7.3 cm^2^), with an absolute difference of 2.4 ± 3.1 cm^2^ and a bias of 0.5 ± 2.4 cm^2^ (SEE = 2.5 cm^2^CCC = 0.951; Fig. 5B, C; Table 2).

Next, estimated ACSA_VL50%_ was used to estimate VL muscle volume using Eqs. 1, 5 and 8, and subsequently compared to measured volume. Muscle volume was on average 558 ± 202 cm^3^ and explained 95% of variance in measured muscle volumes (562 ± 218 cm^3^) with a bias of 1 ± 50 cm^3^ [-97 98 cm^3^ and an absolute difference of 41 ± 27cm^3^ (9.1 ± 7.6% relative difference) vs. measured VL muscle volumes (SEE = 47 cm^3^ [8.4%], CCC = 0.972, Fig. 5D, E; Table 2).

## Discussion

The aim of the present study was to develop a quick, yet accurate, ultrasound-based assessment of the vastus lateralis (VL) muscle volume for application in large-scale studies and clinical practice. To this end, we developed an estimation equation for VL volume, based on a “muscle shape factor”, which describes the shape of the VL along its length, muscle length, and VL anatomical cross-section (ACSA) at 50% muscle length (ACSA_VL50%_). We found that ACSA determines the shape factor that is needed to estimate volume. Validation of our VL volume estimation equation in an independent, heterogeneous cohort achieved high quality estimation accuracy (6.1 ± 5.0% relative difference, *R*^*2*^ = 0.965). We next presented two different approaches to quickly assess ACSA_VL50%_, via ultrasound image stitching, or by estimating ACSA_VL50%_ based on muscle thickness. Both methods performed well in estimating muscle volumes (*R*^*2*^ = 0.91–0.95) with small standard errors of the estimate (SEE = 49–64 cm^3^) and substantial concordance (CCC = 0.95–0.97). Both estimation approaches, particularly the muscle thickness-based method, considerably reduce the measurement complexity and time required to derive muscle volume, bypassing some of the technical difficulties associated with measuring VL muscle volume. Thereby, this approach can be suitable for the application in large-scale research settings and clinical practice.

### An accurate and broadly applicable estimation model of Vastus lateralis muscle volume by ultrasound

The accuracy of using serial ACSAs for assessing muscle volume has been confirmed previously using MRI^15,31^. These studies reported strong correlations (*R*^*2*^ ≥ 0.84) between measured and predicted muscle volume using MRI-based regression models^15,31^. We tested this approach using ultrasound, a cheaper and more widely applicable method, and validated it in an independent sample population. We employed ultrasound images combined with a voxel-interpolation tool to assess muscle volume, which provides a smoother 3D volume compared to the addition of distinct ACSA measurements^15^. Our regression estimates of volume based on measured cross-sectional area differed by 6% from the measured muscle volume, whilst our ultrasound-based estimation model yielded an *R*^2^ of 0.975 and estimated values were within a 5% error margin, performing with similar accuracy to MRI-derived volumes.

Since muscles do not have a cylindrical shape where volume can be calculated by multiplying ACSA with muscle length, a shape factor is essential, adjusting for the non-uniform shape along the muscle length^32^. Our average muscle shape factor of 0.593 was in between previous MRI-derived constants of 0.54 to 0.658^15,25,33,34^ in homogenous populations. We anticipate that these differences are due to orientation of the upper leg during the assessment (completely extended leg during MRI measurements) and differences in participant characteristics (in terms of age and sex-related differences in ACSA and physical fitness). We found our derived muscle shape factor to be dependent on muscle size, with a decrease in muscle shape factor as muscle size increases. Validation of our estimation equation in an independent sample consisting of 35 adults (21–91 years) resulted in highly accurate volume estimates (*R*^*2*^ = 0.965). These findings confirmed the broadly applicable potential of our muscle shape factor analysis across various age groups and muscle sizes. Whether our equation for muscle shape factor also applies to the severely diseased intensive care unit patient^14,35^ remains to be studied.

### Estimation of muscle volume by a single, stitched anatomical cross-section

We measured VL ACSA at 50% muscle length by 2D B-mode ultrasound image stitching as independent input for the estimation of muscle volume, as this was the closest 10% increment to the true maximal ACSA (47 ± 10%). Stitched ACSAs highly correlated with values attained from manually segmented ACSAs in 3D ultrasound image processing software. Previously, the ACSA at 60% femur length (from distal end) was used to obtain an estimation of whole quadriceps volume based on a single ACSA scan^15^. The authors reasoned that this region likely represented the maximal ACSA of both the VL and vastus intermedius, the two largest muscles of the quadriceps, leading to more precise quadriceps volume estimations, albeit an average overestimation of 50cm^3^^15^. However, in our study, maximal ACSA was observed at 47 ± 10% of muscle length in 27 cases. The distance between the distal end of the femur and the end of myotendinous junction of the VL was 3.3 ± 0.9 cm in 25 participants of our cohort, corresponding to ~ 7% of the total muscle length. Our estimation model focusses solely on the VL and not the entire knee extensor muscle group, yet performed accurately, yielding an average absolute difference of 41 ± 27 cm^3^ (9.1 ± 7.6% relative difference) from actual muscle volumes. Additionally, measuring ACSA at 50% muscle length and estimating volume is simple, quick, but still accurate.

Volume estimates derived from stitched ACSAs accounted for 91% of the variance in actual values, showed no proportional bias and an SEE of 10.5%. The entire measurement sequence, including identifying muscle proximal and distal end for determining muscle length, ultrasound imaging, merging and VL ACSA assessment demands approximately 25 min per participant.

### A more simplified and faster ACSA measure for the prediction of muscle volume

To further simplify the measurement sequence for estimating muscle volume we tested a tissue thickness-based estimation of ACSA. Due to the simplifications made, we expected that thickness-based ACSA estimates were less accurate than our measured ACSA or muscle volume. Surprisingly, regression fits of the two methods had a very similar *R*^2^ and standard error of the estimate. While both presented methods can be performed using any ultrasound device, muscle thickness measurements are very fast, with close to no data processing following imaging, requiring 5–10 min per participant to derive an estimation of VL muscle volume. With this considerable reduction in time required to predict muscle volume, bypassing some of the technical difficulties associated with measuring VL muscle volume, plus its degree in accuracy, this approach offers potential for application in large-scale studies and clinical (screening) practice.

### Practical applications

The potential clinical application of the presented models lies in evaluating muscle volumes in both cross-sectional and longitudinal studies focusing on aging^36^,training^28^, disuse and hospitalization^37^, or disease^9^. Many studies often consider muscle thickness or ACSA only as surrogate measure for muscle size and adaptation thereof. However, a muscle may adapt in size by adding or removing sarcomeres in parallel or in series^38^,rendering muscle volume as a more appropriate measure to study skeletal muscle hyper- or atrophy. Franchi et al. investigated whether muscle thickness could serve as a valid marker for MRI-determined ACSA and volume changes following resistance training^22^. Whereas ultrasound-obtained muscle thickness significantly correlated with ACSA and volume before and after 12 weeks of isokinetic resistance training, the association between muscle thickness and volume lost significance when considering relative changes in muscle thickness and volume^22^. Such non-uniform skeletal muscle hypertrophy pattern after resistance training^22^ was also observed in elite sprinters^39^, highlighting the potential heterogeneity in hypertrophic distribution along the length of a muscle belly opposed to regional effects. Our muscle volume estimation model includes muscle length, width, and changes in shape of the muscle along its length. That our muscle shape factor was dependent on muscle size confirmed these findings.

Minor regional alterations in muscle hyper- or atrophy require direct volumetric measurements. As such, the practical utility of our estimation model depends on its sensitivity to detect differences in muscle size. Variations in VL volume among sprinters and non-sprinters (26% difference)^39^, sprint and endurance athletes (23% difference)^40^,and type 2 diabetes mellitus patients and controls (10% difference)^41^ have been reported. Meanwhile, smaller changes in resistance training-induced hypertrophy were found (5.6% difference)^42^. The muscle thickness-based estimation model presented in this study with a standard error of the estimate of 47cm^3^ (8.4%) proves sensitive enough to detect most of the training-related hypertrophy and disease-related atrophy responses above, but not all. Therefore, if concerned with small sample sizes combined with the need of obtaining accurate muscle volume, we recommend the actual measurement of muscle volume (with e.g., MRI or 3D ultrasound), rather than an estimation model with inherent prediction errors. However, when dealing with large clinical cohorts and the clinical meaningful difference is larger than 8.4%, then the assessment time can be significantly reduced by estimating muscle volumes with our estimation model. Therefore, our novel method provides a promising tool to safely, rapidly and accurately quantify VL volume, a crucial lower extremity muscle for power generation, with potential in for instance monitoring critically ill patients with bedside measurements, large randomized controlled trials and application in clinical practice.

### Limitations

Despite its widespread application, ultrasound-based muscle volume estimation encounters challenges inherent in its methodology. Our new estimation method does not replace the current gold standard assessment of muscle size and volume. Variables such as operator proficiency, inter-subject variability in muscle architecture, and the assumptions estimation models are based on may affect their accuracy. Ultrasound-based assessments of muscle size and volume are inherently influenced by probe pressure, alignment, and intra- and inter-operator variability. However, this variability can be minimized through clear instructions and operator expertise. Therefore, the development of this new method should include rigorous training and hands-on experience to enhance consistency and reduce operator-dependent variability. Estimation errors may accumulate during longitudinal monitoring, which limits the applicability for longitudinal studies. Our thickness-based model relies on assumptions (e.g., circular segment geometry) that may not hold in atrophied or pathologically altered muscles. The model assumes that muscle thickness and CSA at a single mid-muscle site are representative of the entire vastus lateralis. However, previous in vivo studies have demonstrated significant intra- and inter-muscular variation in both architecture and cross-sectional area along the quadriceps femoris^43^ and non-uniform CSA distributions within the quadriceps muscle^44^,similar to our heterogeneity in muscle shape factor. Future studies should therefore evaluate whether averaging measures across multiple sites or using extended-field ultrasound reduces this potential source of error. The use of our estimation model in more specific target (clinical) populations such as cachectic patients, those with altered muscle architecture or muscular diseases, especially in longitudinal settings requires further validation.

## Conclusion

In this study, we introduced two approaches for the estimation of vastus lateralis muscle volume, either requiring solely a single anatomical cross-sectional area (ACSA) measure or a single ultrasound image. These approaches significantly decrease the time needed for assessments while maintaining accuracy, thereby enhancing the practicality of ultrasound-based skeletal muscle volume evaluations in populations such as those afflicted by disease, elderly individuals, or trained athletes. Within a 10 min workflow, VL muscle volume can be assessed, albeit with an error of up to 8.4% in identifying changes in muscle size. This technique allows for a safe, cheap, quick, and valid way for quantifying skeletal muscle mass and may be used in medical research, clinical practice, and large-scale studies, particularly when substantial morphological changes are expected. It stands to question in how far these measures perform in more specific target populations such as the cachectic or critically ill patient, especially in longitudinal study settings.

## Data Availability

Data is available upon reasonable request to the corresponding author (RCIW).
